# Supplementary material for: In Vitro and In Silico Wound-Healing Activity of Two Cationic Peptides Derived from Cecropin D in Galleria mellonella
Source: Antibiotics (Basel). 2025 Jun 27;14(7):651. doi: 10.3390/antibiotics14070651 (PMC12291643; doi:10.3390/antibiotics14070651)
Supplement: Supplementary file 1 [file antibiotics-14-00651-s001.zip › antibiotics-3629089-supplementary.pdf]

Supplementary Materials

Figure S1 (Supplementary): Effect of Short and Long Peptides on Cell Migration (In Vitro Wound Closure) at 16 µg/mL for 24 Hours. The table shows the size of the gap: **A**. Untreated cells, **B**. long peptide, and C. short peptide.

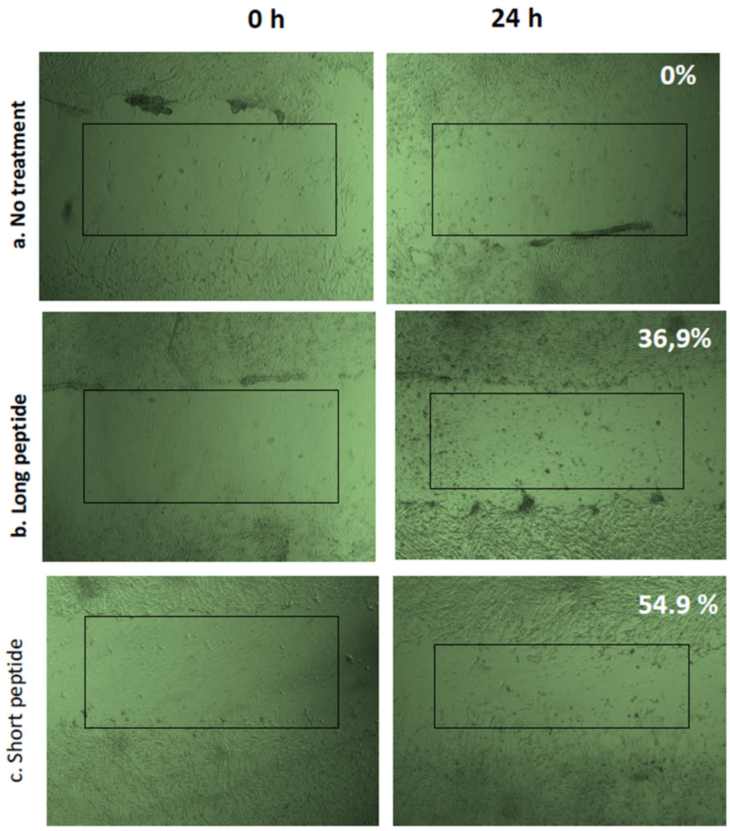

Figure S2 (Supplementary): provides a visual illustration of the differences in docking poses of Short Peptide (A) and Long Peptide (B) in the EGFR receptor binding site. The predicted pose of the Short Peptide (A) facilitates highly favorable interactions, especially robust electrostatic interactions and hydrogen bonds with key receptor residues, such as Asp830 and His850. There are also significant contributions from Pro772, Gln820/849, and Arg962, as highlighted in the energy decomposition analysis (Figure S2A). These interactions with these residues contribute to the Short Peptide's higher observed affinity. In contrast, the pose of the long peptide (B) results in less effective interactions. Although residues such as His850 participate, the same favorable electrostatic interactions are not established with other key acidic/charged residues. Furthermore, the energy analysis (Figure S2B) suggests that residues such as Lys852 in the receptor may have an unfavorable effect on the total binding energy of this peptide. The differences in the positioning of each peptide in relation to these key residues and the charged regions of the pocket visually explain the marked difference in their binding affinities for EGFR.

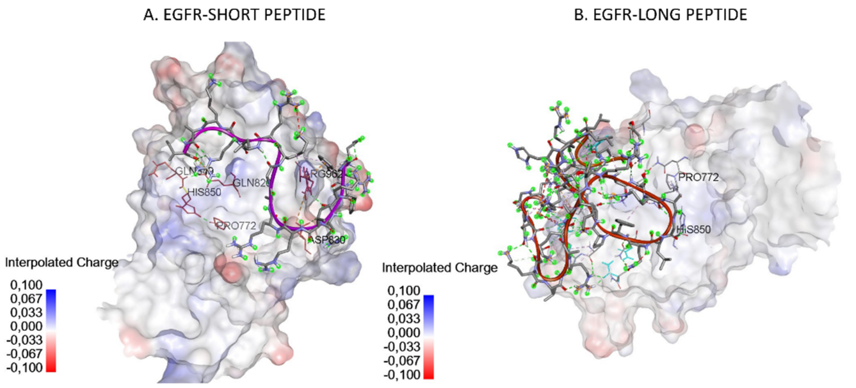

Supplementary Figure S2. Representative Docking Poses of the Short and Long Peptides with the EGFR Receptor. The receptor surface is colored according to the interpolated electrostatic charge (blue for positive and red for negative). For each interaction, the key receptor residues mentioned in Figure S2 are visualized.

Figure S3 shows the predicted molecular docking poses of the short and long peptides with the TGFRβ2 receptor. Both panels show the TGFRβ2 receptor surface colored according to its electrostatic charge (blue for positive and red for negative), revealing the nature of the binding pocket. The peptides are visualized as being bound in the active site, representing the conformations and orientation predicted by docking. Key receptor residues, such as LYS381 and ASP397 in the Short Peptide (A) complex and PHE255 and SER416 in the Long Peptide (B) complex, are highlighted in yellow and participate in binding interactions.

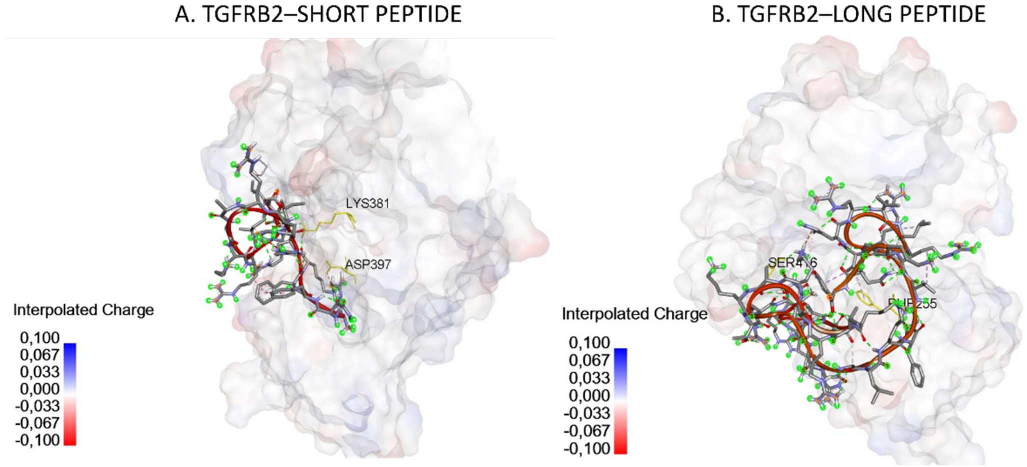

Supplementary Figure S3. Representative Docking Poses of the Short and Long Peptides with the TGFRB2 Receptor. The receptor surface is colored according to the interpolated electrostatic charge (blue for positive and red for negative). Figure S3A highlights the receptor residues LYS381 and ASP397 in yellow. Figure S3B highlights the receptor residues PHE255 and SER416 in yellow.

Figure S4 illustrates the predicted molecular docking poses of the short and long peptides with the VEGFR receptor. In both panels, the surface of the receptor is colored according to the interpolated electrostatic charge (blue for positive, red for negative), indicating the properties of the binding site. The peptides are visualized in their predicted binding poses, showing their conformations within the receptor's active site. Key VEGFR receptor residues that interact with the peptides are highlighted in yellow. These residues include ARG929 and ASP1058 in the Short Peptide (A) complex and ASN923 and ASP1058 in the Long Peptide (B) complex.

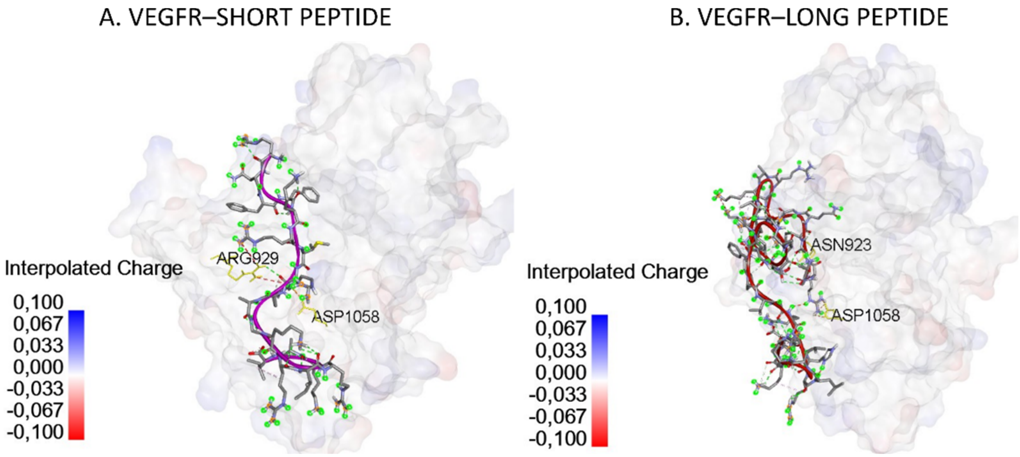

Supplementary Figure S4. Representative Docking Poses of the Short Peptide (A) and the Long Peptide (B) with the VEGFR Receptor. The receptor surface is colored according to the interpolated electrostatic charge (blue for positive, red for negative). In Figure S4A, the receptor residues ARG929 and ASP1058 are highlighted in yellow. In Figure S4B, the residues receptor PHE255 and SER416 are highlighted in yellow.
